# Digital pathology does not improve the prognostic performance of Ki‐67 in a large cohort of penile cancer

**DOI:** 10.1002/2056-4538.70122

**Published:** 2026-11-10

**Authors:** Jan Hrudka, Radoslav Matěj, Michal Grus, Anežka Marková, Zuzana Prouzová, Jan Hojný, Michaela Kendall Bártů, David Čapka, Nicolette Zavillová, Květoslava Michalová, Jiří Soukup, Miroslav Záleský, Petr Waldauf

**Affiliations:** ^1^ Department of Pathology, 3rd Faculty of Medicine Charles University, University Hospital Kralovske Vinohrady Prague Czech Republic; ^2^ Department of Pathology, 1st Faculty of Medicine Charles University, General University Hospital Prague Czech Republic; ^3^ Department of Pathology and Molecular Medicine, 3rd Faculty of Medicine Charles University, Thomayer University Hospital Prague Czech Republic; ^4^ Department of Urology, 3rd Faculty of Medicine Charles University, University Hospital Kralovske Vinohrady Prague Czech Republic; ^5^ Department of Urology, 3rd Faculty of Medicine of Charles University Thomayer University Hospital Prague Czech Republic; ^6^ Šikl´s Institute of Pathology, Faculty of Medicine in Pilsen Charles University, University Hospital Pilsen Pilsen Czech Republic; ^7^ Department of Pathology Military University Hospital Prague Czech Republic; ^8^ The Fingerland Department of Pathology Charles University, Faculty of Medicine in Hradec Králové and University Hospital Hradec Kralove Czech Republic; ^9^ Department of Urology, 1st Faculty of Medicine Charles University, Military University Hospital Prague Czech Republic; ^10^ Department of Anaesthesia and Intensive Care Medicine, 3rd Faculty of Medicine Charles University, University Hospital Kralovske Vinohrady Prague Czech Republic

**Keywords:** penile squamous cell carcinoma, Ki‐67, proliferation index, digital image analysis, interobserver agreement, B lymphocytes, tumour immune microenvironment, prognosis

## Abstract

Penile squamous cell carcinoma (pSCC) is a rare but prognostically unfavourable genitourinary malignancy in which the search for reliable prognostic biomarkers has intensified in recent years. The prognostic role of Ki‐67 in pSCC remains uncertain. This study aimed to assess the prognostic value of Ki‐67 in pSCC using digital image analysis (HALO) and assessment by two pathologists, to compare their prognostic performance and interobserver agreement, and to explore associations with clinicopathological, immune and molecular features. In a large retrospective cohort of 224 invasive pSCCs, Ki‐67 was quantified by digital image analysis and independently estimated by two senior pathologists. Optimal cut‐points (67%, 25%, and 75%) were derived using maximally selected rank statistics. Ki‐67‐high status was significantly associated with 5‐year overall survival with HALO in univariate analysis [hazard ratio (HR) = 1.62, 95% CI 1.03–2.55, *p* = 0.037], and for HALO (HR 1.63, 95% CI 1.03–2.56, *p* = 0.035) and Pathologist 2 (HR 1.63, 95% CI 1.02–2.58, *p* = 0.04) after adjustment for age. When assessed by Pathologist 1, high Ki‐67 expression (≥25%) was significantly associated with shorter cancer‐specific survival. Further covariate‐adjusted analyses showed variable results across assessment methods, with the most consistent prognostic associations observed for Pathologist 2. Penalized spline analysis demonstrated a gradual, approximately linear increase in mortality risk with increasing Ki‐67 values across all three raters, without identifying a clear prognostic threshold. Interobserver agreement was moderate to substantial (Cohen's kappa 0.42–0.61). High Ki‐67 was associated with high grade, p16, and HPV positivity by quantitative PCR (qPCR) in all three raters. Exploratory analyses suggested an inverse association between Ki‐67 and B‐cell/plasma‐cell infiltration. The prognostic value of Ki‐67 in pSCC was non‐robust and depended on the assessment method and chosen cut‐off. Digital image analysis did not significantly outperform manual scoring or improve interobserver agreement. Finally, the inverse association between Ki‐67 and B‐cell/plasma‐cell infiltration represents a potentially novel observation warranting further validation.

## Introduction

Penile squamous cell carcinoma (pSCC) is a relatively uncommon tumour in industrialized countries, with an incidence varying from approximately 0.5 per 100,000 males in the USA to 8.3 per 100,000 males in Brazil, and even more in parts of Africa [1]. Globally, 37,699 new cases were diagnosed in 2022, accounting for 0.2% of all cancers [2]. Among male genitourinary tumours (penis, prostate, testis), pSCC harbours the worst prognosis, particularly in advanced stages [3]. Five‐year survival remains below 70%, with no significant improvement in prognosis over recent decades [3]. According to the WHO, human papillomavirus (HPV)‐induced and HPV‐independent carcinogenic pathways with specific molecular signatures and histological precursors are recognized [4]. Since the early 2020s, intensive research has focused on the development of prognostic markers of penile carcinoma, particularly at the molecular level.

Ki‐67 (antigen Kiel 67) was first described by Gerdes *et al* in 1983 [5]. Ki‐67 is a nuclear protein specific to proliferating cells of mammals and other higher vertebrates, associated with cellular proliferation and ribosomal RNA transcription [6]. Ki‐67 is tightly linked to mitotic chromatin organization and cell‐cycle progression (G1–M phases) in mammalian cells. The protein is absent in resting (G0 phase) cells. Ki‐67‐immunohistochemistry (IHC) is widely used by pathologists to identify proliferation activity. Particularly in cancer tissue, the proportion of Ki‐67–positive tumour cells reflects proliferative activity and has prognostic relevance across multiple cancer types, including SCC and male genitourinary tumours [7, 8, 9, 10, 11]. Nevertheless, the utility of the Ki‐67 proliferation index is limited by poor interobserver agreement among pathologists [12]. To date, most prognostic studies on Ki‐67 in pSCC have focused on manual evaluation of Ki‐67. To the best of our knowledge, the agreement between pathologists and digital image analysis in Ki‐67 IHC assessment has not yet been investigated in penile carcinoma.

The aim of this study was to assess the prognostic value of Ki‐67 in pSCC using automated image analysis, compare its prognostic performance with manual assessment, and explore associations with clinicopathological variables, the tumour immune microenvironment, and DNA mutational profiles derived from our previous studies [13, 14, 15, 16, 17].

## Materials and methods

### Ethics approval and patient consent

This study was approved by the institutional review board and the University Hospital Královské Vinohrady ethics committee, approval number EK‐VP1261012020. The entire research was performed in accordance with the Declaration of Helsinki (https://www.wma.net/policies‐post/wma‐declaration‐of‐helsinki/). Consent to participate was waived due to the retrospective nature of the study.

### Cohort acquisition

A retrospective cohort comprising 224 cases of surgically resected or biopsied and histologically verified invasive pSCC with available formalin‐fixed paraffin‐embedded (FFPE) tumour tissue from 2002 to 2026 was analysed. Follow‐up data were extracted from medical records in the four involved institutions and from the Czech National Oncological Registry. Tissue blocks with representative invasive tumour volume were selected.

### Immunohistochemistry

For IHC, whole slide tissue sections of 4‐μm thickness were stained using the Ventana BenchMark ULTRA autostainer (Ventana Medical Systems, Albuquerque, NM, USA). A mouse monoclonal antibody Ki‐67 (clone MIB‐1; Dako, Glostrup, Denmark, ready to use) was used. The reactions were visualised using the Ultraview Detection System (Ventana Medical Systems) and counterstained with haematoxylin. The stained slides were then dehydrated and mounted in a xylene‐based medium.

### Image analysis and manual slide evaluation

The histological slides were scanned using the Axioscan 7 whole‐slide scanner (Zeiss, Jena, Germany). Whole slide image analysis was performed using the HALO image analysis platform, version 4.2 6399.90 (HALO™, Indica Labs, Albuquerque, NM, USA). The settings for dual stain analysis (blue for haematoxylin, brown for positive nuclear reactivity) are provided in the supplementary material, Table S1. Digital image analysis was performed in hot spots to identify areas with the highest Ki‐67 rate, containing at least 1,000 invasive cancer cells, while avoiding areas with high numbers of tumour infiltrating lymphocytes (TILs). The contribution of TILs nuclear positivity was minimized by adjusting nuclear size thresholds during digital image analysis. The percentage of positively staining cells was recorded. Manual microscopic evaluation was independently performed by two board‐certified pathologists with different professional backgrounds in the same manually marked tumour areas that were subjected to HALO digital image analysis. Both pathologists estimated the percentage of Ki‐67‐positive tumour cells within these areas. Pathologist 1 (RM) is a professor of pathology with more than 25 years of diagnostic experience and broad expertise in oncological and molecular pathology, neuropathology, and pulmonary pathology. Pathologist 2 (JH) is an associate professor of pathology with 15 years of diagnostic experience, specializing in gastrointestinal and urological pathology, with a particular research focus on pSCC.

### Statistical analyses

For overall survival (OS) analysis, Kaplan–Meier analysis was conducted using the log‐rank test, and confidence intervals were calculated using the log–log method. This was followed by a restricted mean survival time analysis with 95% confidence interval (CI). Univariate Cox regressions with 95% CIs were performed to calculate the hazard ratio (HR) for each parameter. Multivariable Cox regression survival analyses were additionally performed adjusting Ki‐67 for age and, in separate models, for each clinicopathological, immunological, HPV, and molecular variable included in the logistic regression/Pearson's chi‐squared analyses described below. Cancer‐specific survival (CSS) analysis was performed using the Aalen–Johansen estimator (cumulative incidence function), distinguishing between penile cancer‐related deaths and deaths from other causes, based on information from clinical documentation and the Czech National Oncological Registry. Survival analyses were truncated at 5 years of follow‐up. The cohort was binarized using the surv_cutpoint function (survminer package 0.5.2), which determines the optimal cut‐point using maximally selected rank statistics from the maxstat R package. Based on the optimal cut‐point, the cohort was classified into Ki‐67‐high and Ki‐67‐low groups. The cut‐point was calculated separately for each rater (HALO image analysis, Pathologist 1, and Pathologist 2). Interobserver agreement (low versus high) between the HALO image analysis results and both pathologists was subjected to Cohen's kappa calculation. The cut‐off value used to dichotomize the cohort for Cohen´s kappa analysis was adopted from the observer who achieved the lowest *p* value (i.e. the highest statistical significance) in the Kaplan–Meier analysis of OS. To avoid arbitrary dichotomization, Ki‐67 was additionally analysed as a continuous variable using a Cox proportional hazards model with penalized splines.

To assess associations of Ki‐67 with clinicopathological variables, logistic regression/Pearson's chi‐squared test was performed. A separate analysis was performed for each rater. The clinicopathological variables were grouped as follows: pT1 + pT2/pT3 + pT4; pNX + pN0/pN1 + pN2 + pN3; grade 1 + 2/grade 3; tumour budding high (≥5 buds/power field) versus low (<5 buds/power field), lymphatic invasion yes/no; vascular invasion yes/no; p16 block positive/non‐block positive, PD‐L1 positive/negative (0 versus 1–100% of tumour cells), B‐cell immunoscore low/high, tumour infiltrating CD20+ cells (low/high), tumour infiltrating CD138+ cells, as described in detail elsewhere [13, 14, 16, 17]. The patients who underwent lymphadenectomy with negative lymph node histology were clustered together with patients without lymphadenectomy (pNX/cN0). The Ki‐67 profiles have been compared with mutational profiles examined by next‐generation sequencing and HPV profiles analysed by qPCR, the results of which and methods have been described by our group elsewhere [15]. Genes altered in ≥10 cases were included in the analysis. All analyses were performed using R version 4.6.1 (R Foundation for Statistical Computing, Vienna, Austria), with survival analyses carried out using the survival package version 3.8.11. A *p* value ≤0.05 was considered statistically significant.

## Results

### Optimal cut‐off values and survival analysis

Research data used in this study are listed in the supplementary material, Table S2. Optimal cut‐off values determined by maximally selected rank statistics were 67%, 25%, and 75% for HALO, Pathologist 1, and Pathologist 2, respectively. OS with univariate and age‐adjusted analysis is summarized in Table 1. Multivariable Cox regression results are summarized in Table 2.

**Table 1:** Kaplan–Meier analysis and univariate and age‐adjusted Cox regression analyses of Ki‐67 in penile squamous cell carcinoma assessed by HALO digital image analysis and two pathologists

Rater	Optimal cut‐point	Ki‐67 low/high *n* = (deaths)	Restricted mean survival time (years)	*p* value (Kaplan–Meier)	Hazard ratio unadjusted	95% CIs	*p* (Cox regression)	Hazard ratio age adjusted	95% CIs	*p* (Cox regression age adjusted)
HALO image analysis	67% (≥)	165 (58)	3.50	**0.035**	1.62	1.03–2.55	**0.037**	1.63	1.03–2.56	**0.035**
		59 (28)	2.93							
Pathologist 1	25% (≥)	31 (9)	3.96	0.091	1.80	0.90–3.59	0.1	1.58	0.79–3.17	0.2
		193 (77)	3.25							
Pathologist 2	75% (≥)	171 (60)	3.47	0.065	1.54	0.97–2.44	0.067	1.63	1.02–2.58	**0.04**
		53 (26)	2.99							

Note the different optimal cut‐points identified for each assessment method. Significant *p* values <0.05 are shown in bold.

**Table 2:** Hazard ratio of Ki‐67 high versus low for 5‐year overall survival (OS), adjusted for age and one further variable at a time

Adjusted for age	*n*	HALO image analysis	Pathologist 1	Pathologist 2
Grade	224	1.53 (0.97–2.41), *p* = 0.068	1.33 (0.65–2.72), *p* = 0.428	1.52 (0.96–2.43), *p* = 0.077
pT stage	221	1.51 (0.96–2.40), *p* = 0.077	1.37 (0.68–2.77), *p* = 0.380	**1.63 (1.02–2.60), *p* = 0.040**
pN stage	224	1.47 (0.93–2.33), *p* = 0.097	1.42 (0.71–2.86), *p* = 0.325	1.52 (0.95–2.42), *p* = 0.079
Lymphatic invasion	223	1.47 (0.93–2.32), *p* = 0.098	1.21 (0.59–2.48), *p* = 0.599	1.46 (0.91–2.33), *p* = 0.113
Venous invasion	223	1.46 (0.93–2.30), *p* = 0.104	1.26 (0.62–2.56), *p* = 0.522	1.40 (0.88–2.24), *p* = 0.158
Tumour budding	220	1.27 (0.80–2.04), *p* = 0.312	1.22 (0.60–2.48), *p* = 0.578	1.32 (0.82–2.12), *p* = 0.258
p16	220	**1.82 (1.14–2.93), *p* = 0.013**	1.92 (0.92–3.98), *p* = 0.081	**1.95 (1.18–3.24), *p* = 0.009**
HPV (qPCR)	131	1.75 (1.00, 3.08) *p* = 0.052	**2.54 (1.04, 6.19), *p* = 0.040**	**2.14 (1.19, 3.84), *p* = 0.011**
PD‐L1	179	**1.64 (1.01, 2.66), *p* = 0.046**	1.76 (0.83, 3.72), *p* = 0.139	**1.80 (1.11, 2.94), *p* = 0.018**
CD138+ invasion front	145	1.49 (0.86, 2.60), *p* = 0.159	1.81 (0.77, 4.21), *p* = 0.171	1.62 (0.93, 2.82), *p* = 0.088
CD20+ tumour centre	145	1.26 (0.73, 2.15), *p* = 0.407	1.91 (0.82, 4.43), *p* = 0.134	1.40 (0.80, 2.44), *p* = 0.236
B‐cell immunoscore	145	1.38 (0.80, 2.37), *p* = 0.244	1.74 (0.75, 4.07), *p* = 0.198	1.55 (0.89, 2.69), *p* = 0.118
HRAS	131	1.59 (0.93, 2.72), *p* = 0.088	2.52 (0.99, 6.44), *p* = 0.053	**1.87 (1.08, 3.23), *p* = 0.025**
TP53	131	**1.90 (1.10, 3.27), *p* = 0.021**	1.78 (0.76, 4.19), *p* = 0.186	**2.49 (1.40, 4.43), *p* = 0.002**
CDKN2A	131	**1.80 (1.05, 3.10), *p* = 0.033**	2.03 (0.87, 4.72), *p* = 0.101	**2.23 (1.27, 3.93), *p* = 0.005**
CHEK1	131	1.43 (0.83–2.47), *p* = 0.194	1.93 (0.82, 4.51), *p* = 0.131	1.68 (0.96, 2.93), *p* = 0.070

Of the 16 adjustment models fitted per rater, Ki‐67 remained significant at *p* < 0.05 in four models for HALO image analysis: 1 for Pathologist 1 and 7 for Pathologist 2. Each cell is a separate Cox model, 95% confidence intervals in parentheses; significant *p* values <0.05 are in bold.

HALO‐assessed high Ki‐67 (*n* = 59) was associated with shorter OS in univariate (HR 1.62, 95% CI 1.03–2.55, *p* = 0.037; Figure 1A) and age‐adjusted analyses (HR 1.63, 95% CI 1.03–2.56, *p* = 0.035). In the additional Cox models, adjusting for age and one further variable at a time, the association remained significant after adjustment for p16 status, PD‐L1 expression, *TP53* and *CDKN2A* mutations. No association with CSS was observed. Both pathologists demonstrated borderline associations with shorter OS in univariate analysis (Pathologist 1: HR 1.80, *p* = 0.10; Figure 1B; Pathologist 2: HR 1.54, *p* = 0.067; Figure 1C). Pathologist 2 reached statistical significance after adjustment for age (HR 1.63, 95% CI 1.02–2.58, *p* = 0.04) and remained significant in models additionally adjusted for pT stage, p16 status, HPV status by qPCR, PD‐L1 expression, and *HRAS*, *TP53*, and *CDKN2A* mutations. When assessed by Pathologist 1, high Ki‐67 expression was significantly associated with shorter CSS (Figure 2). Penalized spline Cox regressions suggested a linear association between Ki‐67 expression and mortality risks, with hazard increasing mainly at high Ki‐67 levels in all three raters, albeit with wide confidence intervals (Figure 1D–F).

**Figure 1:**
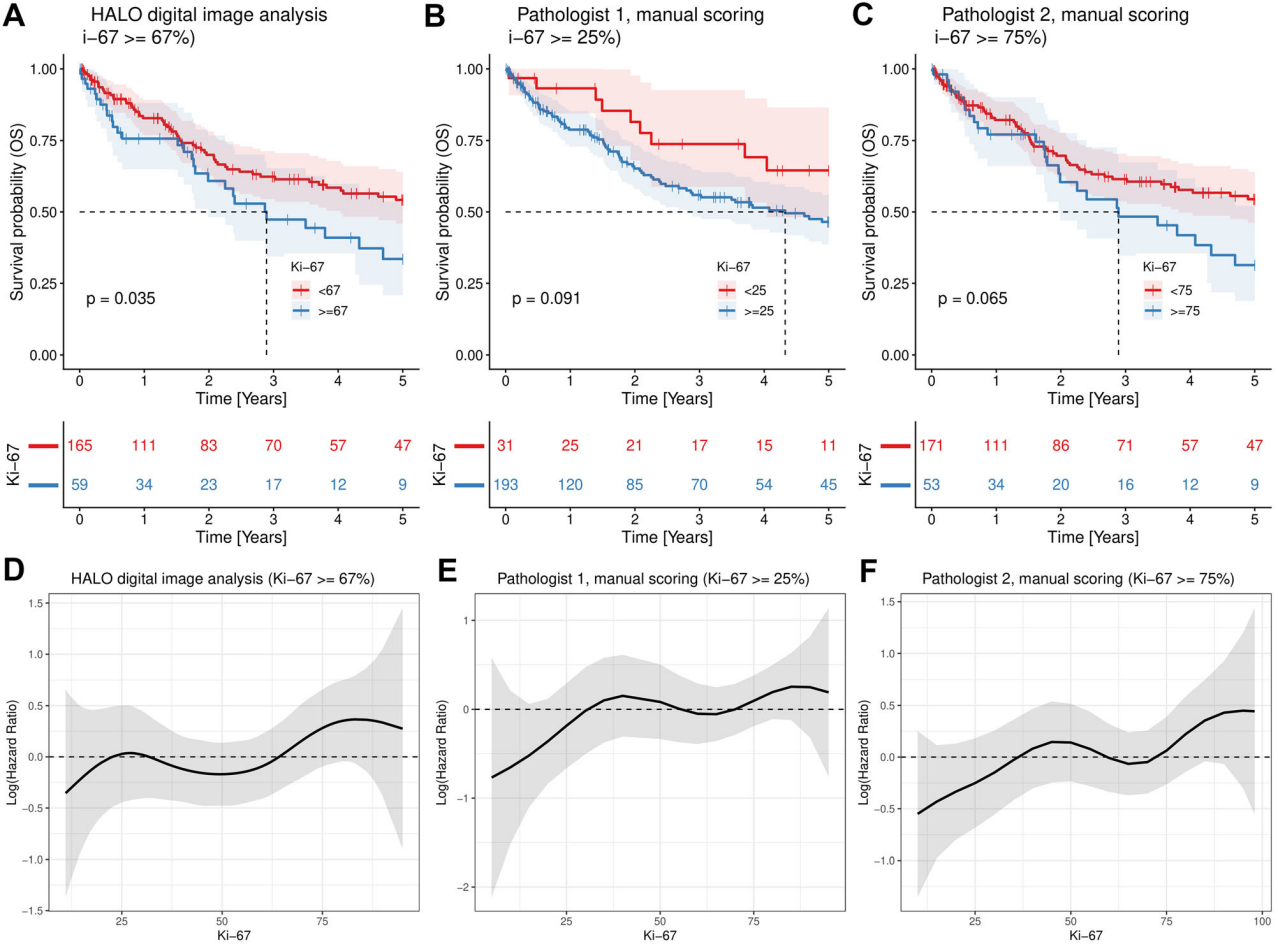
Ki‐67 expression and 5‐year overall survival. (A–C) Kaplan–Meier curves demonstrating 5‐year OS according to Ki‐67 status assessed by (A) HALO digital image analysis, (B) Pathologist 1, and (C) Pathologist 2, using their respective optimal cut‐offs. (D–F) Relationship between Ki‐67 expression and 5‐year mortality risk, modelled as a continuous variable using a Cox proportional hazards model with penalized splines. Higher Ki‐67 values were associated with a gradual increase in mortality risk, although the confidence intervals were wide. The spline curves suggest an approximately linear relationship between Ki‐67 expression and mortality in all three raters (HALO image analysis, Pathologist 1, and Pathologist 2), without evidence of a distinct prognostic threshold. Created with ggplot2 package in R software.

**Figure 2:**
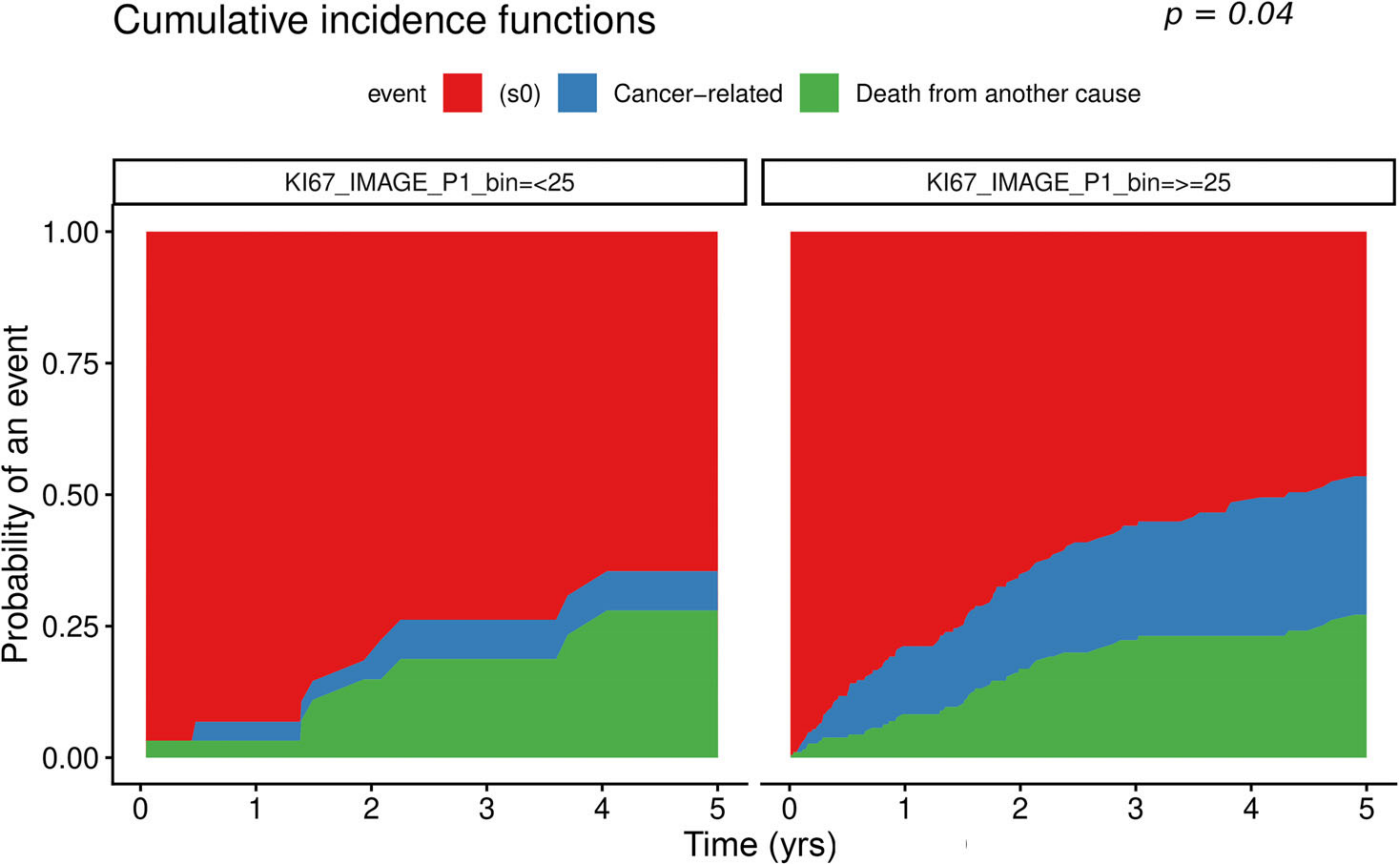
Aalen–Johansen cumulative incidence functions for cancer‐specific death according to Ki‐67 expression assessed by Pathologist 1 using the optimal cut‐off of 25%, *p* value rendered by Gray's test. High Ki‐67 expression was associated with a significantly higher cumulative incidence of penile cancer‐related death (*p* = 0.04), while deaths from other causes were treated as competing events. Created with ggplot2 package in R software.

### Analysis of associations

#### Associations of Ki‐67 with clinicopathological, immunological, and molecular variables

Logistic regression/chi‐squared test results are summarized in Table 3. High Ki‐67 was consistently associated with grade 3, p16 overexpression, and HPV positivity by qPCR across all three assessment methods. Two of the three raters also demonstrated associations with PD‐L1 positivity and lower B‐cell‐related immune measures, including lower intratumoural CD20+ cell density and a lower B‐cell immunoscore. Associations with advanced pathological features (tumour budding and lymphatic invasion), individual immune parameters, and specific gene alterations were less consistent across assessment methods. Associations with individual gene alterations were observer‐dependent: *TP53* mutation was associated with high Ki‐67 by HALO, whereas *HRAS* mutation was associated with low Ki‐67 according to Pathologist 1 and *CDKN2A* mutation with low Ki‐67 according to Pathologist 2. Associations with advanced pT stage, nodal stage, and venous invasion did not reach statistical significance.

**Table 3:** Associations of Ki‐67 with clinicopathological, immunological, and molecular variables according to HALO digital image analysis and two pathologists

	Not assessed	Ki‐67 HALO image analysis, cut‐off 67% (≥)	Ki‐67 Pathologist 1, cut‐off 25% (≥)	Ki‐67 Pathologist 2, cut‐off 75% (≥)
Grade 1 + 2 (*n* = 157) versus 3 (*n* = 67)	0	OR = 2.62, 95% CI 1.34–5.14, ***p* = 0.003**	OR = 7.32, 95% CI 1.76–65.3, ***p* = 0.001**	OR = 1.98, 95% CI 0.98–3.95, ***p* = 0.04**
pT stage 1 + 2 (*n* = 183) versus 3 + 4 (*n* = 38)	3	OR = 1.84, 95% CI 0.81–4.08, *p* = 0.11	OR = 3.38, 95% CI 0.79–30.5, *p* = 0.12	OR = 1.65, 95% CI 0.69–3.75, *p* = 0.2
N stage pNX/cN0 + pN0 (*n* = 182) versus pN1 + 2 + 3 (*n* = 42)	0	OR = 1.98, 95% CI 0.91–4.26, *p* = 0.079	OR = 3.78, 95% CI 0.89–34.0, *p* = 0.08	OR = 1.37, 95% CI 0.59–3.07, *p* = 0.4
Lymphatic invasion absent (*n* = 154) versus present (*n* = 69)	1	OR = 1.81, 95% CI 0.92–3.52, *p* = 0.07	OR = 4.86, 95% CI 1.42–25.9, ***p* = 0.006**	OR = 1.49, 95% CI 0.74–2.98, *p* = 0.2
Venous invasion absent (*n* = 188) versus present (*n* = 35)	1	OR = 2.11, 95% CI 0.91–4.77, *p* = 0.06	OR = 6.42, 95% CI 0.99–271, *p* = 0.058	OR = 1.35, 95% CI 0.53–3.19, *p* = 0.5
Tumour budding <5 buds/PF (*n* = 127) versus ≥5 buds/PF (*n* = 93)	4	OR = 2.13, 95% CI 1.11–4.14, ***p* = 0.019**	OR = 1.95, 95% CI 0.81–5.08, *p* = 0.12	OR = 1.75, 95% CI 0.89–3.47, *p* = 0.11
p16 block positive (*n* = 120) versus non‐block positive + negative (*n* = 100)	4	OR = 2.94, 95% CI 1.47–5.88, ***p* = 0.001**	OR = 14.29, 95% CI 4.17–100, ***p* < 0.001**	OR = 3.33, 95% CI 1.61–7.14, ***p* < 0.001**
HPV (qPCR) negative (*n* = 67) versus positive (*n* = 64)	93	OR = 3.76, 95% CI 1.61–9.23, ***p* = 0.001**	OR = 24.5, 95% CI 3.64–1,047, ***p* < 0.001**	OR = 4.37, 95% CI 1.69–12.4, ***p* < 0.001**
PD‐L1 negative (*n* = 44) versus TPS ≥1% (*n* = 135)	45	OR = 2.39, 95% CI 0.99–6.45, ***p* = 0.04**	OR = 3.81, 95% CI 1.42–10.3, ***p* = 0.004**	OR = 1.89, 95% CI 0.77–5.13, *p* = 0.2
CD138+ invasion front <median (*n* = 67) versus ≥median (*n* = 78)	79	OR = 0.33, 95% CI 0.15–0.74, ***p* = 0.004**	OR = 0.75, 95% CI 0.25–2.15, *p* = 0.6	OR = 0.45, 95% CI 0.19–1.03, *p* = 0.053
CD20+ tumour centre <median (*n* = 71) versus ≥median (*n* = 74)	79	OR = 0.45, 95% CI 0.20–0.99, ***p* = 0.045**	OR = 0.83, 95% CI 0.28–2.39, *p* = 0.8	OR = 0.38, 95% CI 0.16–0.89, ***p* = 0.02**
B‐cell immunoscore 0 + 1 (*n* = 49) versus 2–4 (*n* = 96)	79	OR = 0.4, 95% CI 0.18–0.89, ***p* = 0.02**	OR = 0.45, 95% CI 0.10–1.52, *p* = 0.2	OR = 0.4, 95% CI 0.17–0.93, ***p* = 0.025**
HRAS wild type (*n* = 120) versus mutated (*n* = 11)	93	OR = 0.2, 95% CI 0–1.51, *p* = 0.2	OR = 0.04, 95% CI 0.01–0.21, ***p* < 0.001**	OR = 0.29, 95% CI 0.01–2.21, *p* = 0.3
TP53 wild type (*n* = 99) versus mutated (*n* = 32)	93	OR = 7.41, 95% CI 1.25–78.4, ***p* = 0.012**	OR = ∞, 95% CI 0.3–∞, *p* = 0.6	OR = 3.35, 95% CI 0.59–19.2, *p* = 0.1
CDKN2A wild type (*n* = 102) versus mutated (*n* = 29)	93	OR = 0.42, 95% CI 0.13–1.19, *p* = 0.085	OR = 3.31, 95% CI 0.72–31.1, *p* = 0.2	OR = 0.15, 95% CI 0.02–0.68, ***p* = 0.005**
CHEK1 wild type (*n* = 123) versus mutated (*n* = 8)	93	OR = 0.50, 95% CI 0.15–1.42, *p* = 0.2	OR = 1.16, 95% CI 0.33–5.21, *p* > 0.9	OR = 0.29, 95% CI 0.05–1.07, *p* = 0.052

Overall cohort, *n* = 224. Odds ratios and 95% confidence intervals from logistic regression/Pearson's chi‐squared test; the odds ratio compares the second group with the first, so values above 1 mean the second group is more often Ki‐67‐high. Ki‐67 cut‐offs: HALO 67%, Pathologist 1 25%, and Pathologist 2 75%. Patients in whom a variable was not assessed are excluded from the corresponding odds ratio. Significant associations (*p* < 0.05) are shown in bold.

#### Inter‐rater agreement analysis

The optimal cut‐point was determined for HALO image analysis using the maximally selected rank statistics (function surv_cutpoint, package survminer). This cut‐point was then applied uniformly to all three measurement methods – HALO image analysis, Pathologist 1, and Pathologist 2 – to classify patients into Ki‐67‐low (below cut‐point) and Ki‐67‐high (at or above cut‐point) groups. The cut‐point determined by HALO (67%) was selected as the reference value for inter‐rater agreement analysis as it yielded the lowest *p* value in the survival analysis. The results of applying the common cut‐point across all three measurement methods are shown in Table 4.

**Table 4:** Number of Ki‐67‐low and Ki‐67‐high penile cancer cases according to cut‐point 67% derived from HALO providing the lowest *p* value in survival analysis

Rater	*n*	Low Ki‐67 (<67)	High Ki‐67 (≥67)
HALO image analysis	224	165	59
Pathologist 1	224	126	98
Pathologist 2	224	146	78

Cohen's kappa (*κ*) was calculated for each pair of raters using binary Ki‐67 classifications (Ki‐67‐low/Ki‐67‐high) based on the common cut‐point of 67%, derived from HALO. Confidence intervals were calculated using asymptotic standard errors. Fleiss' kappa was calculated across all three raters using Ki‐67 values dichotomised at the common cut‐point of 67%. Complete concordance among all three assessment methods was observed in 151/224 (67.4%) cases, including 107 (47.8%) Ki‐67‐low and 44 (19.6%) Ki‐67‐high tumours (Figure 3A,B). Discordance was present in 73 (32.6%) cases, most commonly reflecting disagreement between HALO and both pathologists (36/73, 49.3%), whereas isolated disagreement between the two pathologists with concordant HALO classification occurred less frequently (15/73, 20.5%). Complete disagreement among all three raters was not observed. The kappa calculation results are summarised in Table 5.

**Figure 3:**
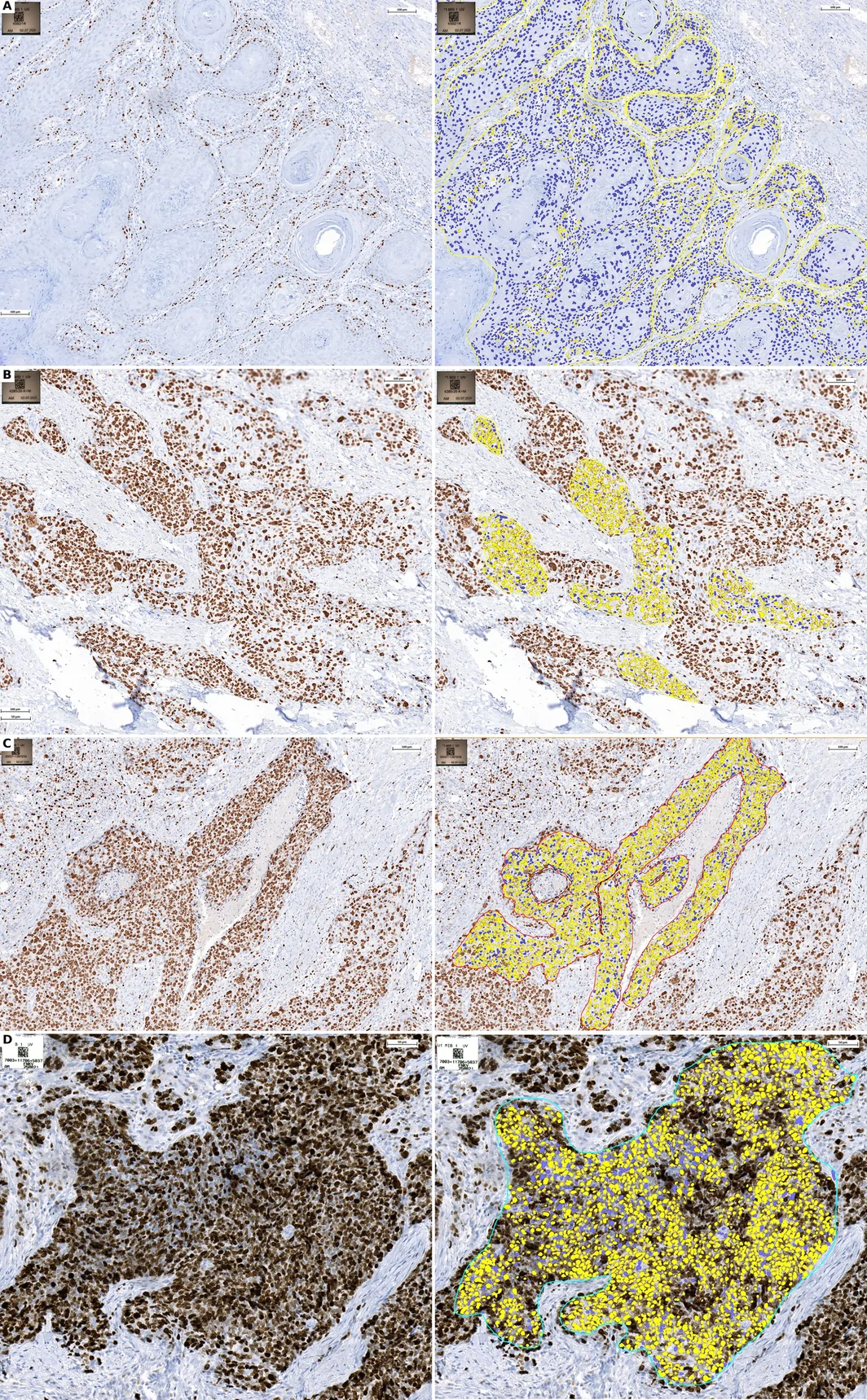
Representative images of Ki‐67 immunohistochemistry in penile squamous cell carcinoma (left) and the corresponding HALO digital image analysis with automated identification of Ki‐67‐positive nuclei (right). The positive nuclei are highlighted in yellow and negative nuclei in blue. (A) Example of a tumour consistently classified as Ki‐67‐low by all three raters: 10% (Pathologist 2), 15% (HALO), and 25% (Pathologist 1). (B) Example of a tumour consistently classified as Ki‐67‐high by all three raters: 70% (Pathologist 2), 75% (Pathologist 1), and 77% (HALO). (C) Example of a tumour classified as Ki‐67‐high by both pathologists but as Ki‐67‐low by HALO: 80% (Pathologist 1), 75% (Pathologist 2), and 65% (HALO). (D) Example of a tumour classified as Ki‐67‐low by both pathologists but as Ki‐67‐high by HALO: 60% (Pathologist 1), 60% (Pathologist 2), and 86% (HALO). Created with HALO image analysis platform, version 4.2 6399.90 (HALO™, Indica Labs, Albuquerque, NM, USA).

**Table 5:** Cohen's kappa (*κ*) and Fleiss' kappa calculation documenting moderate agreement between HALO image analysis and both pathologists and between all three raters, and substantial agreement between both pathologists

Comparison	*n*	Kappa	*z*	*p*	95% CI (asymptotic)	Interpretation
HALO versus Pathologist 1	224	0.421	6.784	<0.001	0.299–0.543	Moderate
HALO versus Pathologist 2	224	0.552	8.424	<0.001	0.423–0.68	Moderate
Pathologist 1 versus Pathologist 2	224	0.61	9.294	<0.001	0.482–0.739	Substantial
All three raters	224	0.522	13.54	<0.001	Not applicable	Moderate

Pairwise comparison of continuous Ki‐67 values is demonstrated in Table 6. Pathologist 1 tended to assign higher Ki‐67 values, whereas HALO more frequently generated the lowest estimates (Figure 3C,D). Substantial agreement (*κ* = 0.6–0.8) [18] was achieved only in comparison of Pathologists 1 and 2.

**Table 6:** Directional comparison of continuous Ki‐67 values between HALO digital image analysis and manual assessment

Comparison	Higher, *n* (%)	Equal, *n* (%)	Lower, *n* (%)
HALO versus Pathologist 1	76 (33.9)	9 (4.0)	139 (62.1)
HALO versus Pathologist 2	100 (44.6)	6 (2.7)	118 (52.7)
Pathologist 1 versus Pathologist 2	112 (50.0)	38 (17.0)	74 (33.0)

Higher = first rater assigned a higher Ki‐67 value than the second rater.

## Discussion

Previous studies evaluating the prognostic role of Ki‐67 in pSCC have yielded inconsistent results. Early studies associated high Ki‐67 expression with high tumour grade and lymph node metastases [19, 20, 21], although the latter was not confirmed in our cohort despite several previous reports [19, 20, 22]. None of the published studies, including the most recent ones, demonstrated a consistent association between Ki‐67 and survival outcomes [23]. In this largest published pSCC series evaluating Ki‐67 to date, its association with OS varied according to the assessment method and covariate adjustment, whereas Pathologist 1 also demonstrated an association with CSS. Overall, these findings indicate that the prognostic performance of Ki‐67 is non‐robust and observer‐dependent.

Penalized spline Cox regression suggested an approximately linear relationship between Ki‐67 expression and mortality risk, with lower Ki‐67 values associated with lower mortality risk and vice versa. This survival modelling was intentionally used to avoid information loss caused by arbitrary dichotomization. The increase in hazard across the continuum of Ki‐67 values was gradual and accompanied by broad confidence intervals. Collectively, these findings suggest that highly proliferative tumours tend to behave more aggressively, although no biologically robust prognostic threshold could be identified (e.g. a distinct ‘low‐risk’ versus ‘high‐risk’ Ki‐67 value).

Human observers and digital image analysis achieved comparable levels of statistical significance (*p* values between 0.03 and 0.1), but at markedly different optimal cut‐offs (from 25% to 75%). Taken together, our findings argue against the routine clinical use of Ki‐67 as a robust prognostic biomarker in pSCC. More broadly, our results raise the question of whether currently used Ki‐67 cut‐offs in other tumour types should also be interpreted with caution. Several recent studies used spline‐based modelling of Ki‐67 in colorectal [24] and breast [25] cancer, which questioned the existence of universal biologically meaningful cut‐offs. Ahlin *et al* used arbitrary 10% interval cut‐offs in breast cancer and demonstrated very similar *p* values for metastasis‐free survival at the 50%, 70%, and 80% thresholds [26]. Tashima *et al* showed that optimal Ki‐67 cut‐points differed across breast cancer subtypes defined by hormone receptor and HER2 status [27].

A general limitation of Ki‐67 assessment is its limited reproducibility across different observers and assessment methods. Several studies have reported substantial agreement between manual and digital Ki‐67 assessment in breast cancer [28, 29, 30, 31] and neuroendocrine neoplasia [32, 33, 34], in which Ki‐67 quantification constitutes a mandatory component of tumour grading. Studies reporting poor agreement between manual and automated Ki‐67 assessment have also been published in neuroendocrine neoplasms [35]. Moderate agreement was observed in our penile carcinoma cohort. The better agreement reported in breast carcinoma and neuroendocrine neoplasms compared with our cohort may be partly explained by differences in the distribution of Ki‐67 values. Pathologists may be more accurate in distinguishing relatively low proliferation indices (e.g. 1% versus 5% or 10% versus 20%) than high proliferation rates (e.g. 60% versus 70%), corresponding to the prognostic cut‐off identified in our cohort.

In addition to inter‐method agreement, comparison of the prognostic information of Ki‐67 assessed by different methods is also of interest. Dawe *et al* reported improved prognostic performance using a deep‐learning–based Ki‐67 scoring approach compared with manual assessment in breast cancer [36]. In contrast, HALO‐based digital image analysis in our study did not clearly outperform human observers, suggesting that digital pathology alone may not overcome the fundamental challenges associated with Ki‐67 evaluation. Although digital analysis is often perceived as objective, its performance still depends on user‐defined parameters, including calibration of staining intensity thresholds and image segmentation settings. The two pathologists demonstrated better agreement with each other than with HALO. This may be partly explained by the superior ability of human observers to distinguish Ki‐67‐positive immune cells from tumour cells during manual assessment. Limited reproducibility of Ki‐67 assessment may not solely reflect observer‐related factors. Considerable variability in staining intensity and chromogenic signal has been documented for Ki‐67. This variation may affect outputs across different slides in both manual and automated quantification [35]. Importantly, neither manual nor digital assessment can currently be regarded as an unquestionable gold standard. Both approaches are influenced by methodological and technical factors, making determination of the ‘true’ Ki‐67 value inherently challenging.

Beyond its prognostic impact, high Ki‐67 expression was associated with several clinicopathological, immunological, and molecular features. All three raters consistently demonstrated significant associations with grade 3, p16 overexpression and HPV positivity determined by qPCR. In line with our results, some studies described higher Ki‐67 expression in HPV‐associated tumours based on PCR analysis [19, 22], which was also observed in our study using both HPV qPCR and p16 IHC as a surrogate marker of HPV infection [37]. An association between PD‐L1 expression and high Ki‐67 was observed with HALO and Pathologist 1, but not Pathologist 2. De Bacco *et al* also demonstrated an association between p16 expression, Ki‐67 proliferation index, and PD‐L1 positivity in pSCC [38]. The association between high Ki‐67 expression and high histological grade is not unexpected, as both reflect increased proliferative activity [20]. The relationship between p16/HPV status and PD‐L1 expression remains controversial, with published studies reporting conflicting results [14, 38, 39, 40]. The association between Ki‐67 and HPV positivity is in line with the study by Protzel *et al*, who demonstrated a correlation between strong Ki‐67 expression, HPV DNA detection, and the presence of inguinal lymph node metastases [19]. The association between HPV positivity and high Ki‐67 expression is biologically plausible, as HPV E6 and E7 oncoproteins disrupt the p53 and Rb pathways, promote cell‐cycle progression, inhibit apoptosis, and lead to sustained cellular proliferation [41, 42].

In the HALO assessment, high Ki‐67 expression was also linked to tumour budding, a well‐established adverse prognostic factor that has recently gained attention in pSCC [13, 43, 44], although this association was not reproduced by either pathologist. Tumour budding has previously been shown to be strongly associated with an abnormal p53 phenotype and is characteristic of the aggressive subgroup of HPV‐independent, *TP53*‐altered pSCCs [13, 44, 45]. Interestingly, TP53‐mutated tumours showed higher Ki‐67 expression in the HALO assessment, although this association was not reproduced by either pathologist. Increased proliferative activity may be one of the biological features contributing to the aggressive behaviour of TP53‐altered HPV‐independent pSCC. An intriguing finding was the association between low Ki‐67 expression and *HRAS* mutation. In pSCC, *HRAS* alterations occur almost exclusively in HPV‐independent tumours [15, 46] and have previously been associated with low risk histopathological features [15]. Although observed only with Pathologist 1 assessment, the association between *HRAS* mutation and low Ki‐67 is consistent with our previous observation that *HRAS*‐mutated pSCCs tend to display low‐risk histopathological features. Our study also suggested an inverse association between high Ki‐67 expression and B‐cell/plasma‐cell infiltration, most consistently observed with HALO and Pathologist 2 assessments. While anti‐tumour immunity has become a major focus of contemporary cancer research, B cell–mediated immune responses have historically received considerably less attention than T cell immunity. B cells and plasma cells play a critical immunomodulatory role in melanoma [47], breast [48, 49, 50], colorectal [51], kidney [52], liver [53], and lung cancer [54]. In most of these, increased B‐cell and plasma‐cell infiltration has been associated with enhanced anti‐tumour immune responses and more favourable clinical outcomes [55]. We have previously reported similar findings in pSCC, where increased B‐cell and plasma‐cell infiltration and the presence of tertiary lymphoid structures were associated with better prognosis [16]. Direct associations between Ki‐67 expression and B‐cell/plasma‐cell–mediated immunity have not been systematically studied in pSCC, and remain poorly explored in other malignancies. In contrast to our findings, Löfdahl *et al* reported a weak but positive correlation between immune cell infiltration, including CD20‐positive B cells, and the Ki‐67 proliferation index in breast carcinoma [56]. While Ki‐67 reflects tumour‐intrinsic proliferative activity, B cells and plasma cells are markers of the host anti‐tumour immune response. Our findings suggest that highly proliferative penile carcinomas may be associated with a relatively B‐cell‐depleted immune microenvironment, but require future validation and mechanistic investigation, for example by transcriptomic analyses.

The present study has several limitations. Part of the observed interobserver variability may reflect differences in defining the threshold for Ki‐67 positivity, particularly in nuclei exhibiting weak IHC staining. Such borderline‐positive nuclei may be inconsistently classified during manual assessment. An analogous limitation applies to digital image analysis, where positivity thresholds must likewise be manually predefined. Finally, the manual assessment was performed by only two pathologists with different professional backgrounds, which may limit the generalizability of the interobserver findings.

## Conclusion

Although high Ki‐67 expression was associated with worse survival and selected clinicopathological features, including high grade, its prognostic performance was not robust and depended on the assessment method and selected cut‐off. Digital image analysis did not substantially improve interobserver agreement or provide better prognostic stratification than expert manual assessment. At the same time, exploratory analyses suggested an inverse association between high Ki‐67 expression and B‐cell/plasma‐cell infiltration, representing a potentially novel biological observation that warrants further investigation. Collectively, these findings argue against the routine use of Ki‐67 as a robust prognostic biomarker in pSCC and suggest that future studies should explore the integration of proliferative, molecular, and immune parameters rather than relying on isolated Ki‐67 thresholds.

## Author contributions statement

JHr designed the study, performed the digital analysis, interpreted the data and wrote the manuscript. MG and AM performed the digital analysis and interpreted the data. ZP performed the histopathologic examination. RM gathered the study cohort, performed the histopathologic examination, supervised the research and reviewed the manuscript. JHo performed the molecular analysis and reviewed the manuscript. MKB gathered the study cohort, searched the follow‐up data, and reviewed the manuscript. DČ searched for the follow‐up data. NZ gathered the study cohort, examined the B‐cell associated variables and searched for the follow‐up data. KM, JS and MZ gathered the study cohort and searched for the follow‐up data. PW performed the statistical analysis, supervised the research and reviewed the manuscript. All authors read and approved the final version of the manuscript.

## Supporting information


**Table S1.** HALO image analysis software settings for dual stain analysis

**Table S2.** Research dataset used in the present study, including patient age, survival time and status, clinicopathological characteristics, immunohistochemical and molecular data, and Ki‐67 proliferation indices assessed by HALO digital image analysis and independently by two pathologists (Pathologist 1 and Pathologist 2) (provided as a separate Excel file)

## Data Availability

The survival, immunohistochemical, clinicopathological data, and Ki‐67 proliferation indices are presented in the supplementary material, Table S2. Molecular data are available on request (corresponding author).
