# Supplementary material for: Digital pathology does not improve the prognostic performance of Ki‐67 in a large cohort of penile cancer
Source: J Pathol Clin Res. 2026 Nov 10;12(6):e70122. doi: 10.1002/2056-4538.70122 (PMC13749154; doi:10.1002/2056-4538.70122)
Supplement: Supplementary file 1 [file CJP2-12-e70122-s002.pdf]

Digital pathology does not improve the prognostic performance of Ki-67 in a large cohort of penile cancer

J Hrudka *et al. J Pathol Clin Res* https://doi.org/10.1002/2056-4538.70122

Supplementary Table S1

Supplementary Table S2 is provided as a separate Excel file

Table S1. HALO image analysis software settings for dual stain analysis.

<AlgorithmSettings AlgorithmName="Indica Labs - Multiplex IHC v4.2.2" Version="1.0"><Parameter Name="Class List" Value="" /><Parameter Name="Classifier" Value="" /><Parameter Name="Classifier Output Type" Value="Mask" /><Parameter Name="Classifier Pipeline" Value="" /><Parameter Name="Classify Registered" Value="" /><Parameter Name="Image Zoom" Value="1" /><Parameter Name="Membrane Masker" Value="" /><Parameter Name="Membrane Masker Type" Value="None" /><Parameter Name="Nuclear Masker" Value="Nuclei Seg (Plugin) - BF v1.0.0" /><Parameter Name="Nuclear Masker Type" Value="AI Custom" /><Parameter Name="Phenotyper" Value="" /><Parameter Name="Phenotyper Filter" Value="" /><Parameter Name="Phenotyper Segmentation Type" Value="" /><Parameter Name="num_markers" Value="2 stains" /><Parameter Name="use_nn" Priority="1" Value="false" /><Parameter Name="nuclear_contrast" Priority="2" Value="0.5" /><Parameter Name="nuclear_min_intensity" Priority="3" Value="0.053" /><Parameter Name="nuclear_min_area" Priority="4" Value="11.3" /><Parameter Name="nuclear_max_area" Priority="5" Value="1000" /><Parameter Name="nuclear_roundness" Priority="6" Value="0" /><Parameter Name="nuclear_segg_agg" Priority="7" Value="0" /><Parameter Name="nuclear_fill_holes" Priority="8" Value="false" /><Parameter Name="cyto_radius" Priority="9" Value="0" /><Parameter Name="membrane_segg_agg" Priority="10" Value="0" /><Parameter Name="cell_min_area" Priority="11" Value="10" /><Parameter Name="cell_max_area" Priority="12" Value="600" /><Parameter Name="phenotypes" Priority="13" Value="{"Phenotypes":[]}" /><Parameter Name="min_tissue_od" Priority="14" Value="0" /><Parameter Name="tissue_edge_thickness" Priority="15" Value="0" /><Parameter Name="verbose" Priority="16" Value="false" /><Parameter Name="stain_name" Index="0" Priority="17" Value="Stain 1" /><Parameter Name="Stain 1" Index="0" Priority="18" Value="0.587,0.691,0.862" /><Parameter Name="stain_nuclei_weight" Index="0" Priority="19" Value="1" /><Parameter Name="stain_use_membrane" Index="0" Priority="20" Value="False" /><Parameter Name="stain_markup_color" Index="0" Priority="21" Value="255,0,0" /><Parameter Name="stain_nuclei_positive_threshold_weak" Index="0" Priority="22" Value="0.22321428571428573" /><Parameter Name="stain_nuclei_positive_threshold_mod" Index="0" Priority="23" Value="2.5" /><Parameter Name="stain_nuclei_positive_threshold_strong" Index="0" Priority="24" Value="2.5" /><Parameter Name="stain_nuclei_completeness_threshold" Index="0" Priority="25" Value="51.7" /><Parameter Name="stain_cyto_positive_threshold_weak" Index="0" Priority="26" Value="2.5" /><Parameter Name="stain_cyto_positive_threshold_mod" Index="0" Priority="27" Value="2.5" /><Parameter Name="stain_cyto_positive_threshold_strong" Index="0" Priority="28" Value="2.5" /><Parameter Name="stain_cyto_completeness_threshold" Index="0" Priority="29" Value="0" /><Parameter Name="stain_membrane_positive_threshold_weak" Index="0" Priority="30" Value="2.5" /><Parameter Name="stain_membrane_positive_threshold_mod" Index="0" Priority="31" Value="2.5" /><Parameter Name="stain_membrane_positive_threshold_strong" Index="0" Priority="32" Value="2.5" /><Parameter Name="stain_membrane_completeness_threshold" Index="0" Priority="33" Value="0" /><Parameter Name="stain_name" Index="1" Priority="34"

Value="Stain 2" /><Parameter Name="Stain 2" Index="1" Priority="35" Value="0.335,0.303,0.236" /><Parameter Name="stain_nuclei_weight" Index="1" Priority="36" Value="1" /><Parameter Name="stain_use_membrane" Index="1" Priority="37" Value="False" /><Parameter Name="stain_markup_color" Index="1" Priority="38" Value="117,126,148" /><Parameter Name="stain_nuclei_positive_threshold_weak" Index="1" Priority="39" Value="2.5" /><Parameter Name="stain_nuclei_positive_threshold_mod" Index="1" Priority="40" Value="2.5" /><Parameter Name="stain_nuclei_positive_threshold_strong" Index="1" Priority="41" Value="2.5" /><Parameter Name="stain_nuclei_completeness_threshold" Index="1" Priority="42" Value="0" /><Parameter Name="stain_cyto_positive_threshold_weak" Index="1" Priority="43" Value="2.5" /><Parameter Name="stain_cyto_positive_threshold_mod" Index="1" Priority="44" Value="2.5" /><Parameter Name="stain_cyto_positive_threshold_strong" Index="1" Priority="45" Value="2.5" /><Parameter Name="stain_cyto_completeness_threshold" Index="1" Priority="46" Value="0" /><Parameter Name="stain_membrane_positive_threshold_weak" Index="1" Priority="47" Value="2.5" /><Parameter Name="stain_membrane_positive_threshold_mod" Index="1" Priority="48" Value="2.5" /><Parameter Name="stain_membrane_positive_threshold_strong" Index="1" Priority="49" Value="2.5" /><Parameter Name="stain_membrane_completeness_threshold" Index="1" Priority="50" Value="0" /><Parameter Name="exclusion_stain" Priority="998" Value="None" /><Parameter Name="output_image" Priority="999" Value="Stain 1 Markup" /></AlgorithmSettings>
